# Embelin Improves the Spatial Memory and Hippocampal Long-Term Potentiation in a Rat Model of Chronic Cerebral Hypoperfusion

**DOI:** 10.1038/s41598-019-50954-y

**Published:** 2019-10-10

**Authors:** Saatheeyavaane Bhuvanendran, Siti Najmi Syuhadaa Bakar, Yatinesh Kumari, Iekhsan Othman, Mohd. Farooq Shaikh, Zurina Hassan

**Affiliations:** 1 Neuropharmacology Research Laboratory, Jeffrey Cheah School of Medicine and Health Sciences, Monash University Malaysia, Bandar Sunway, Selangor Malaysia; 2 Centre for Drug Research, Universiti Sains Malaysia, Penang, Malaysia; 3 Brain Research Institute, Jeffrey Cheah School of Medicine and Health Sciences, Monash University Malaysia, Selangor, Malaysia

**Keywords:** Alzheimer's disease, Molecular medicine

## Abstract

Alzheimer’s disease (AD) is the second most occurring neurological disorder after stroke and is associated with cerebral hypoperfusion, possibly contributing to cognitive impairment. In the present study, neuroprotective and anti-AD effects of embelin were evaluated in chronic cerebral hypoperfusion (CCH) rat model using permanent bilateral common carotid artery occlusion (BCCAO) method. Rats were administered with embelin at doses of 0.3, 0.6 or 1.2 mg/kg (i.p) on day 14 post-surgery and tested in Morris water maze (MWM) followed by electrophysiological recordings to access cognitive abilities and synaptic plasticity. The hippocampal brain regions were extracted for gene expression and neurotransmitters analysis. Treatment with embelin at the doses of 0.3 and 0.6 mg/kg significantly reversed the spatial memory impairment induced by CCH in rats. Embelin treatment has significantly protected synaptic plasticity impairment as assessed by hippocampal long-term potentiation (LTP) test. The mechanism of this study demonstrated that embelin treatment alleviated the decreased expression of BDNF, CREB1, APP, Mapt, SOD1 and NFκB mRNA levels caused by CCH rats. Furthermore, treatment with embelin demonstrated neuromodulatory activity by its ability to restore hippocampal neurotransmitters. Overall these data suggest that embelin improve memory and synaptic plasticity impairment in CCH rats and can be a potential drug candidate for neurodegenerative disease-related cognitive disorders.

## Introduction

Alzheimer’s disease (AD) is the second most common neurological disorder and is associated with cerebral hypoperfusion. Recognised as the second cause of age related cognitive deficits after Alzheimer’s disease(AD), vascular dementia (VD) is also considered as a neurological disorder^1^. The hypothesis on vascular dementia proposed that the reduction in blood flow to the brain affects the glial and neuronal cells energy demands, thus causing neurodegeneration and brain dysfunction^2^. It has been discovered that this age-related neurodegenerative disorder contributed to 20% of all dementia patients, which is foreseen to be tripled by 2050^3,4^. The decrease in cerebral blood flow namely chronic cerebral hypoperfusion (CCH) has been detected in cerebrovascular patients who later develop marked cognitive deficiency^5^.

In this study, a rodent model that mimics the CCH was induced using permanent bilateral common carotid arteries occlusion (BCCAO) method. According to Damodaran, *et al*.^6^, BCCAO rats are suitable as CCH model since they are able to cause a significant decrease in cerebral blood flow by 32% in the hippocampus and by 21% in the cortex^7^. For the duration of four days to three months (chronic phase), these rats demonstrated learning and memory impairments^6^ followed by neuronal damage and oxidative stress, which resemble the deficiencies that occur during dementia in humans^8,9^. Studies based on this animal model revealed the potential strategies to thwart, slow down and reverse the neurodegenerative disease progression associated with imbalance cerebral blood flow^10^.

Synaptic integrity and plasticity are crucial for a healthy brain function especially in learning and memory^11^. Contrary, cognitive decline symptoms in neurological disorders including AD and dementia have been associated with synaptic plasticity impairment^12^ by evidence of loss of synapse numbers and functions in the hippocampus^13,14^. Recently, numerous studies on long-term potentiation (LTP) have emerged as it is considered an indicator of synaptic plasticity at the cellular level that correlates with changes in cognitive function^13^. Experimental evidences have reported that by assessing long-term potentiation (LTP), neural plasticity dysfunction can be directly detected^11^ as LTP inhibition has been observed in the CA1 region of the hippocampal in CCH rat models^15,16^.

Embelin is a promising benzoquinone compound (C_17_H_26_O_4_) with a molecular weight of 294.39 g/mol belonging to the fruits of *Embelia ribes* Burm (Family: Myrsinaceae)^17^. In Indian traditional medicinal practice, the fruits of *Embelia ribes* are consumed as a brain tonic to cure disorders related to central nervous system (CNS)^18^. Kundap, *et al*.^19^, stated that the anticonvulsant, antidepressant and anxiolytic activities possessed by embelin have been demonstrated by many studies with the ability to improve neurological-related disorders such as Huntington’s disease, multiple sclerosis, sickness behaviour, ischemia and traumatic brain injury. Recently, the neuroprotective effect of embelin in Alzheimer’s disease-like condition model has been reported^17,20^, but specific neuroprotective mechanism of action against memory impairments in CCH-induced BCCAO rats remain elusive.

Thus, this study aims at identifying whether the administration of embelin can pharmacologically ameliorate the memory impairment in BCCAO rats. The first part of the experiment involved the behavioural effect of embelin on spatial cognitive performance using the Morris water maze (MWM), which was followed by the assessment on the effects of embelin on LTP in the CA1 (Cornu ammonis 1) region of the hippocampus using *in vivo* electrophysiological recording. LTP of embelin in this study is the first to be reported, which may reveal the synaptic plasticity properties of this yellowish-orange compound as a potential therapy for Alzheimer’s disease-like conditions. On the other hand, the last part of this experiment involved the extraction of rat’s brain for studying their gene expression and neurotransmitter to aid in determining the potential mechanism responsible for the neuroprotective effect of embelin in vascular cognitive impairment and dementia conditions.

## Results

### Effects of embelin on memory performance in MWM test in CCH-induced rats

**Figure 1:**
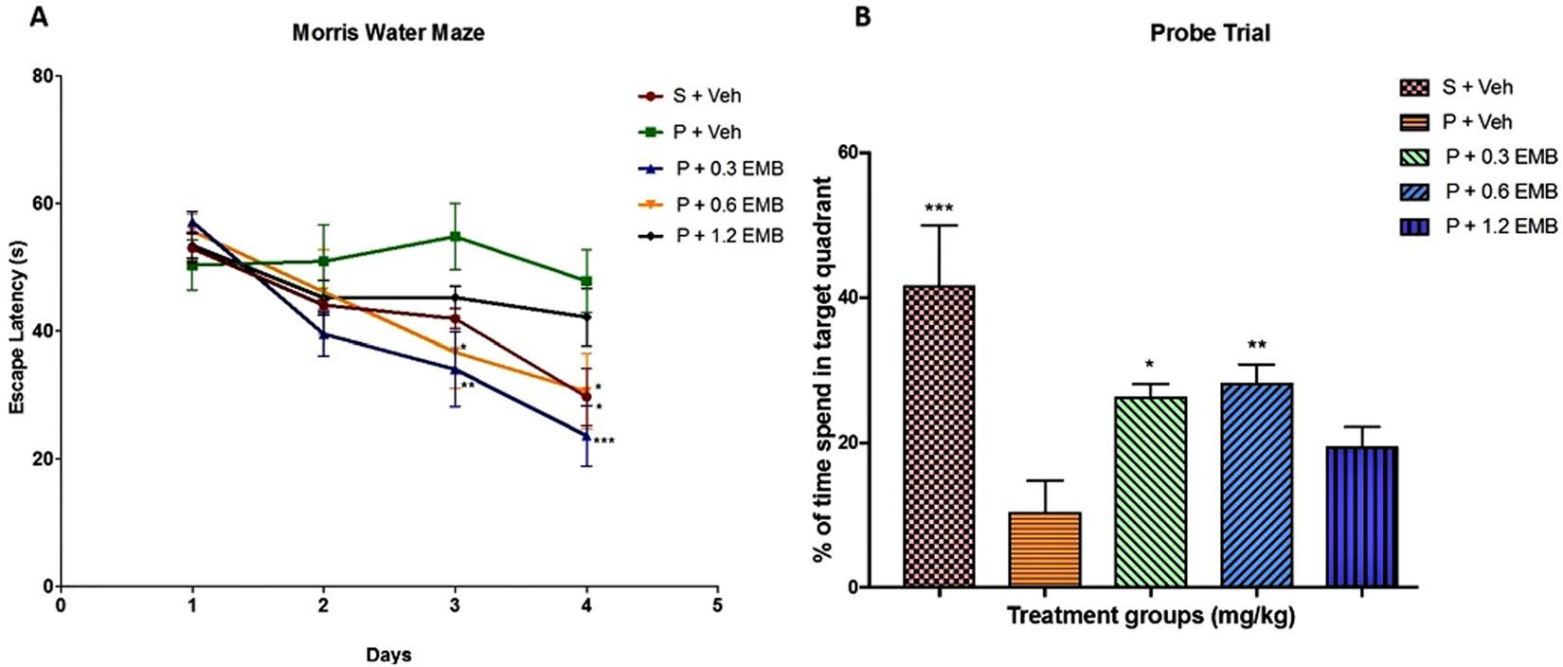
The effect of post-training administration of embelin on the performance of MWM in BCCAO- induced CCH rats. (**A**) Escape latency in the MWM test of each training day. (**B**) Mean time spent on the platform zone in the MWM test. The behavioral analysis for (**A**,**B**) were compared to negative control group (P + Veh). Data are expressed as mean ± S.E.M. from (n = 7) with *p < 0.05, **p < 0.01, ***p < 0.001.

The MWM test was used in this study to evaluate the effects of embelin on the spatial memory impairment induced by CCH rats. Embelin was administered after each training session to study the post-training effect of this compound on learning and memory functions among BCCAO rats. Figure 1A displays the swim traces of 5 groups on each test day where it was apparent that the BCCAO rats treated with only vehicle (negative control) demonstrated longer latency to reach the submerged platform than that of the sham rats (p = 0.0108), thus indicating a poor learning disability following the BCCAO surgery. Nevertheless, significant differences were confirmed by two-way repeated ANOVA analysis between escape latency and post-training administration of embelin due to treatment groups (F_4,86_ = 5.541; p = 0.0005) and training day (F_3,86 = _20.50; p < 0.0001). The interaction between embelin post-training treatment groups and traning day was statistically significant (F_12,86_ = 2.065; p = 0.0277) with the post hoc analysis. Besides, a gradual shortening of latency during the training stage in BCCAO was noticed on rats with embelin, which showed significant enhancement with 0.3 mg/kg embelin on training day 3 (p = 0.0016) and day 4 (p = 0.0007). Moreover, a significant decrease in escape latency was recorded in BCCAO rats when receiving 0.6 mg/kg embelin on day 3 (p = 0.0175) and 4 (p = 0.0258). However, the group with 1.2 mg/kg embelin displayed a decrease in escape latency on day two, but it was not significant as the performance to find the hidden platform remained constant for day 3 (p = 0.3392) and 4 (p > 0.9999) even after the training.

On the other hand, the untreated BCCAO rats were seen unable to recall the location of platform in the probe trial on the fifth day, thus spending significantly less time in target quadrant compared to that of sham rats (p = 0.0001) (Fig. 1B). A significant difference in post-training treatments with embelin (F_4,19_ = 8.477; p = 0.0004) was noted between the groups in the percentage time spent in the target quadrant during the probe trial. BCCAO rats with embelin of 0.3 mg/kg (p = 0.0116) and 0.6 mg/kg (p = 0.0068) after the training session significantly spent longer time in the target quadrant compared to untreated BCCAO rats, which indicates memory improvement. However, there was no significant difference in probe trial task on rats receiving 1.2 mg/kg embelin than those with BCCAO (p = 0.2393).

### LTP in the CA3-CA1 region of the hippocampus

**Figure 2:**
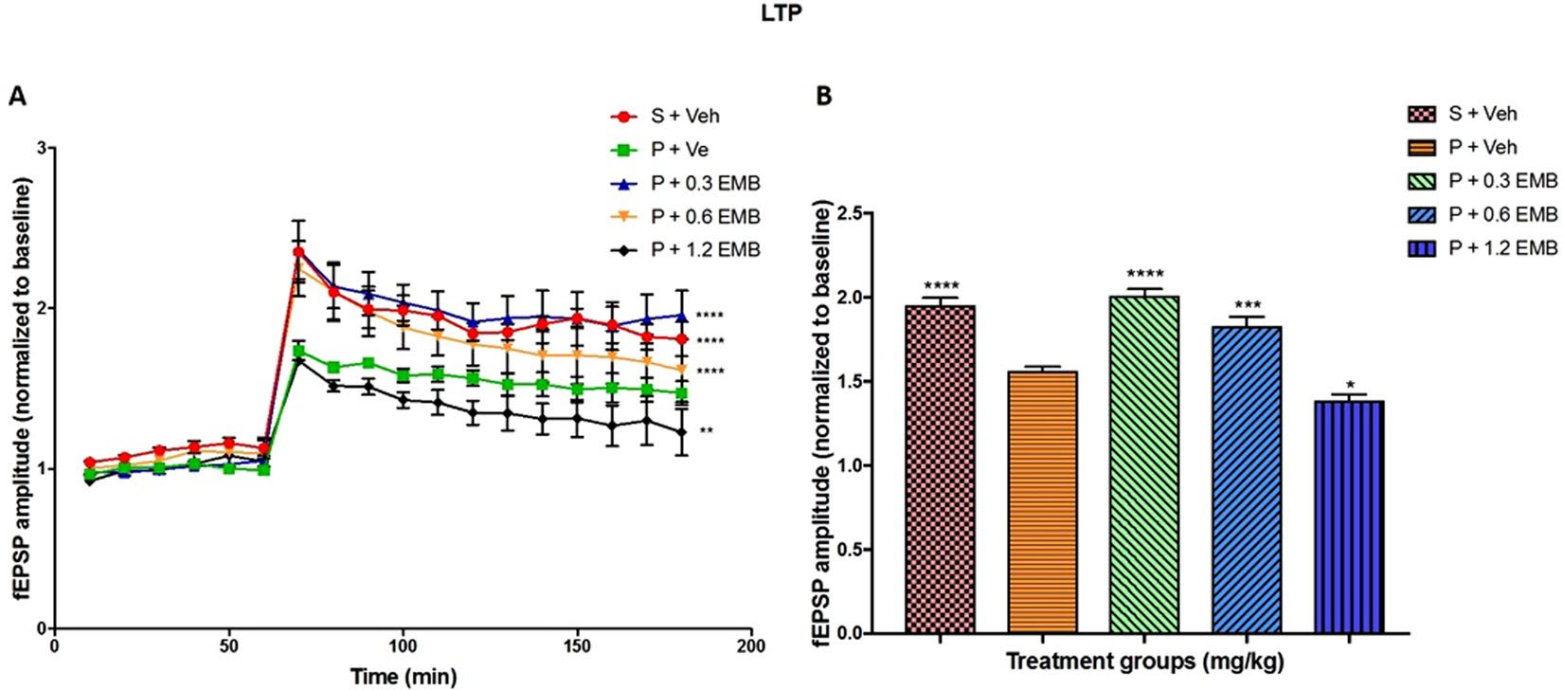
The effect of embelin on LTP in the CA1 hippocampus. (**A**) Time course changes in normalized fEPSP amplitude after TBS. (**B**) The mean fEPSP amplitude during the 2-h interval after TBS. The analysis for (**A**,**B**) were compared to negative control group (P + Veh). Data are expressed as the mean ± S.E.M. (n = 6) with *p < 0.05, **p < 0.01, ***p < 0.001.

LTP recording was performed to investigate the synaptic plasticity in the CA3-CA1 region of the hippocampus. The normalised time course changes of fEPSP amplitude to one hr baseline period are presented in Fig. 2A showing that the fEPSP amplitude of all the five groups was increased after TBS and stabilised to different levels above the baseline period. Meanwhile, two-way ANOVA analysis confirmed a significant difference in the synaptic activity in hippocampus due to treatment (F_4, 288_ = 70.20; p = 0.0001) and time (F_17, 288_ = 60.64; p = 0.0001) as well as the interaction between treatment and time (F_68, 288_ = 1.884; p = 0.0002). The results indicated reduced LTP in the BCCAO group treated with vehicle alone compared to that of the sham group (p < 0.0001) with enhanced LTP in the BCCAO rats treated with embelin 0.3 and 0.6 mg/kg groups (p < 0.0001). Statistical mean values of the fEPSP amplitude for the last 2 hrs after TBS are graphically represented in Fig. 2B. It was discovered that after high-frequency stimulation, LTP formation in the hippocampus has significantly inhibited BCCAO group treated with vehicle (1.57 ± 0.02) than those in the sham group (1.96 ± 0.04; p < 0.0001). In addition, one-way ANOVA showed significant restoration of the LTP inhibition in the vehicle group with 0.3 mg/kg (2.01 ± 0.04; p < 0.0001) and 0.6 mg/kg (1.83 ± 0.06; p = 0.001) embelin in BCCAO rats. Interestingly, 1.2 mg/kg embelin treated BCCAO (1.38 ± 0.04; p = 0.0136) group displayed a significant decrease in fEPSP amplitude after TBS compared to the BCCAO group.

### Changes in the mRNA level in the hippocampus by Real-time PCR

**Figure 3:**
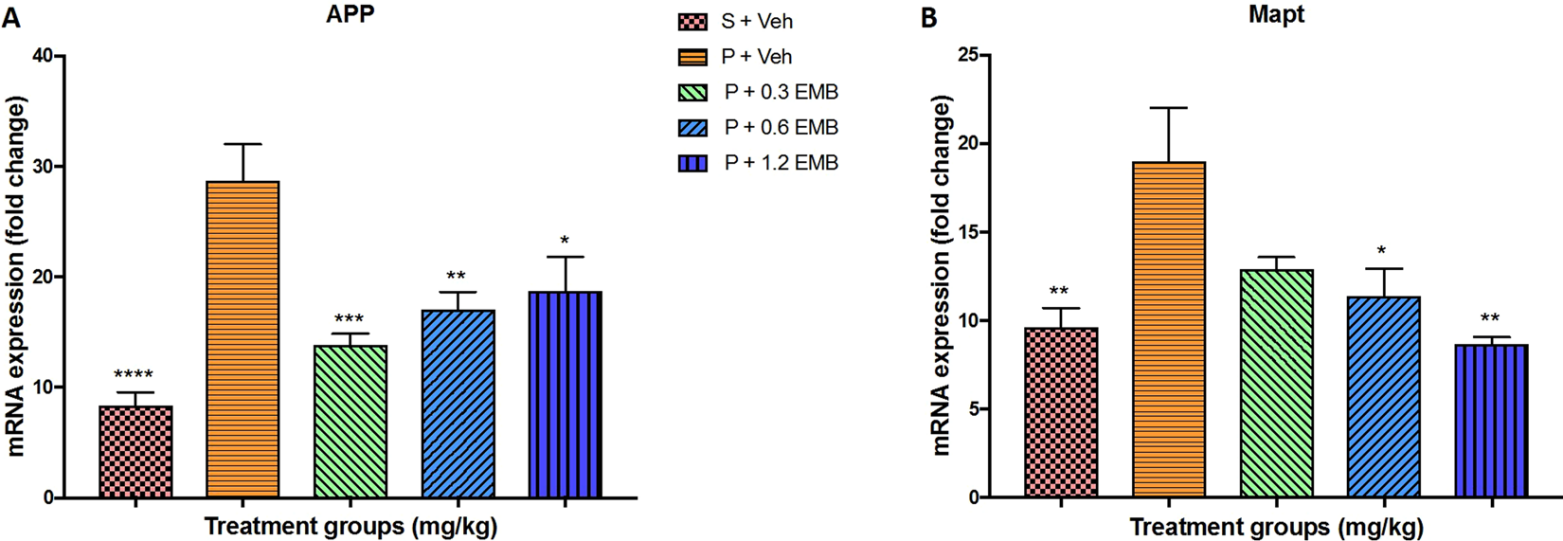
The effect of embelin on amyloid-beta (**A**) APP and Tau (**B**) Mapt mRNA expression level in the rat hippocampus using real-time PCR. All changes in the expression levels were compared to negative control group (P + Veh). Data are expressed as the mean ± S.E.M. (n = 6) with *p < 0.05, **p < 0.01, ***p < 0.001, ****p < 0.001.

The expression of mRNA in BCCAO rat hippocampal tissues after embelin treatment was studied by Real-time PCR analysis showing a significant increase in APP mRNA expression of BCCAO group (p < 0.0001) than sham rats. In addition, a significant reduction in APP mRNA expression was observed in BCCAO rats treated with 0.3 mg/kg (p = 0.0008) and 0.6 mg/kg (p = 0.0049) embelin compared to BCCAO group. Meanwhile, group with 1.2 mg/kg embelin also demonstrated a significant reduction but a slight increase in APP mRNA expression compared to other embelin groups (p = 0.0225) (Fig. 3A). Moreover, the Mapt mRNA expression was seen increasing significantly for BCCAO rats treated with vehicle alone compared to sham-vehicle treated group (p = 0.0046). BCCAO rats that were treated with embelin at dose 0.6 mg/kg (p = 0.0126) and 1.2 mg/kg (p = 0.0012) had a significant reduction in Mapt mRNA expression as compared to BCCAO group. However, BCCAO rats treated with 0.3 mg/kg embelin also shown reduction in Mapt mRNA expression but it was not significant (p = 0.0512). Figure 3B graphically displays the expression level of Mapt mRNA for each rat group.

**Figure 4:**
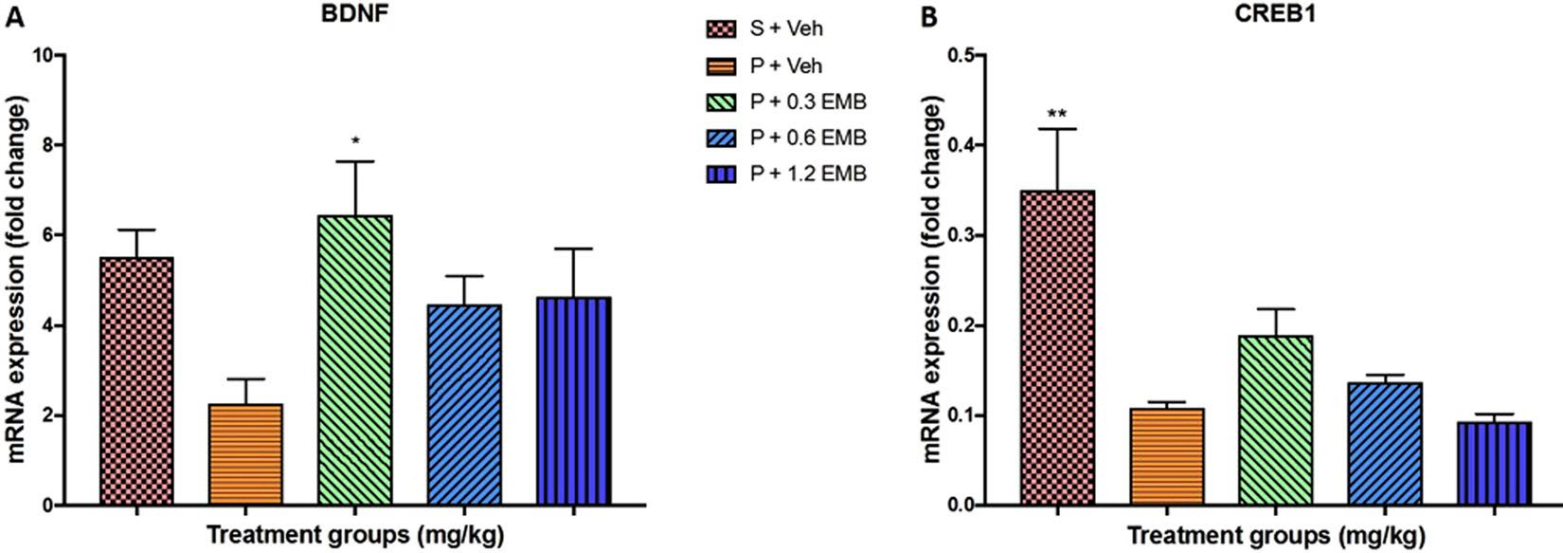
The effect of embelin on synaptic plasticity mRNA expression level (**A**) BDNF and (**B**) CREB1 in the rat hippocampus using real-time PCR. All changes in the expression levels were compared to negative control group (P + Veh). Data are expressed as the mean ± S.E.M. (n = 6) with *p < 0.05, **p < 0.01, ***p < 0.001.

**Figure 5:**
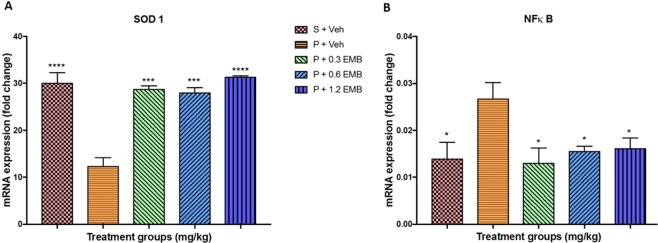
The effect of embelin on oxidative stress (**A**) SOD1 and neuroinflammation (**B**) NF-κB mRNA expression level in the rat hippocampus using real-time PCR. All changes in the expression levels were compared to negative control group (P + Veh). Data are expressed as the mean ± S.E.M. (n = 6) with *p < 0.05, **p < 0.01, ***p < 0.001, ****p < 0.001.

On top of that, the expression of CREB 1 mRNA level was observed significantly decreasing in BCCAO rats than sham-vehicle treated rats (p = 0.020). However, statistically non-significant change in the gene expression level of CREB 1 for all the embelin treated groups was recorded compared to BCCAO group at a level of ∗α = 0.05. Despite this, Fig. 4B graphically presents that the CREB 1 expression by the 0.3 and 0.6 mg/kg embelin treated groups had visibly increased compared to the BCCAO-vehicle treated alone group. On the other hand, non-significant downregulation of BDNF mRNA level was noted in BCCAO group compared with sham groups where only 0.3 mg/kg of embelin treated BCCAO rats presented a significant increase in BDNF mRNA expression (p = 0.0232). Besides, there was a non-significant increase in BDNF mRNA expression level in 0.6 mg/kg (p = 0.3867) and 1.2 mg/kg (p = 0.3053) of embelin groups when compared to BCCAO group as shown in Fig. 4A. Furthermore, Fig. 5A depicts a significant depletion of the gene level of SOD1 in terms of antioxidant gene expression for the BCCAO group compared to the sham-vehicle treated group (p < 0.0001). Additionally, all embelin treated BCCAO rats demonstrated a significant increase in SOD1 expression with 3-fold change (p < 0.0001) compared to BCCAO group. On the other hand, there was a substantial rise in the gene expression level of NFκB for BCCAO group compared to that of sham-vehicle treated rats (p = 0.0248). The mRNA level of NFκB was significantly ameliorated by embelin treatment in 0.3 mg/kg (p = 0.0157), 0.6 mg/kg (p = 0.0347) and 1.2 mg/kg (p = 0.0496) of embelin groups when compared to BCCAO group as presented in Fig. 5B.

### Estimation of neurotransmitter levels in the hippocampus by LC-MS/MS

**Figure 6:**
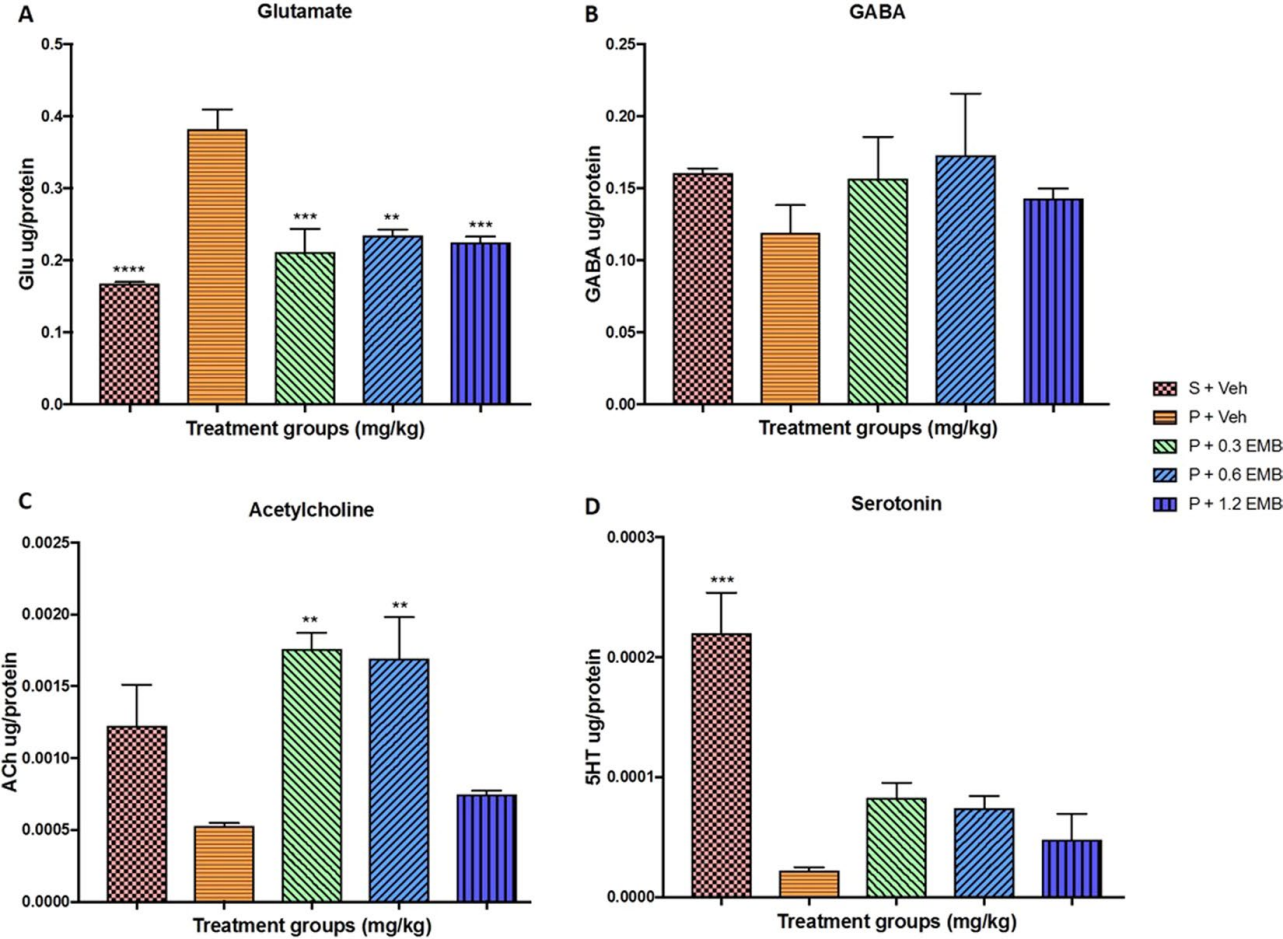
The effect of embelin on hippocampus neurotransmitters level of CCH-induced BCCAO rats. The neurotransmitters included are (**A**) Glutamate (**B**) GABA (**C**) Acetylcholine (**D**) Serotonin. All changes in the expression levels were compared to negative control group (P + Veh). Data are expressed as the mean ± S.E.M. (n = 6) with *p < 0.05, **p < 0.01, ***p < 0.001, ****p < 0.001.

CCH-induced BCCAO rats administered with vehicle caused a significant increase in the level of Glu (p < 0.0001) compared to sham rats. BCCAO rats treated with embelin in 0.3 mg/kg (p = 0.0002), 0.6 mg/kg (p = 0.0024) and 1.2 mg/kg (p = 0.0006) groups significantly ameliorated the levels of Glu compared to BCCAO vehicle-treated group. On the other hand, levels of GABA, ACh, and 5HT in the hippocampal of BCCAO rats were reduced in comparison to sham control group. However, only 5HT levels were detected to be significantly less in BCCAO rats at a p-value = 0.0001. Nevertheless, embelin treatment has restored the decreased levels of GABA and 5HT compared to BCCAO vehicle-treated rats, but were not significant. Interestingly, BCCAO rats treated with embelin at dose 0.3 mg/kg (p = 0.0026) and 0.6 mg/kg (p = 0.0041) significantly attenuated the decrease in the level of ACh compared to that of BCCAO group. Meanwhile, embelin at dose 1.2 mg/kg group failed to restore the decreased level of ACh caused by CCH-induced BCCAO rats as can be seen in Fig. 6.

## Discussion

The therapeutic effects of embelin were found promising in many neurological-related disorders using various animal models^19^. Recently, embelin treatment in rodents has successfully reversed scopolamine^17^ and streptozotocin-induced cognitive deficits^20^ by modulating the antioxidant pathway, cholinergic activity, hippocampal neurogenesis and neuroinflammatory cytokines. Even though embelin has been found as a potential molecule in the previous findings against AD-like conditions, to date, no studies have reported the memory-improving effects of embelin in a CCH animal model. Thus, this paper is the first to report the acute effects of embelin in cognitive impairment and pathophysiological transformation following two weeks of carotid arteries occlusion. The selection of dose range for embelin post-training treatment used in this current study was determined based on previously published study^17^.

According to Farkas, *et al*.^9^, the cerebral blood flow to hippocampus was reduced by ~60% compared to control level, which progressively continued for a week with their effects remained for several months resulting from the BCCAO surgery. Hence, BCCAO-induced CCH in rodents could be extrapolated to human cerebral hypoperfusion resembling ageing or demented people^21^. Those with AD tend to have deficits in spatial abilities as they become lost in familiar places and unable to relocate the place^22^. Therefore, MWM has been considered as a standard method to study the spatial cognitive function in rats^23^. In the current study, it was discovered that the BCCAO group showed longer escape latency during the 4-days training period, as well as during probe trials when the platform was removed from the pool. These data suggest that the BCCAO rats had significantly impaired hippocampus-dependent task that were in line with previous studies^6,24^. In contrast to BCCAO group, rats treated with embelin 0.3 and 0.6 mg/kg groups were able to significantly locate the platform faster and spent more time in the target quadrant. In the present study, embelin was administered after the training session to demonstrate the effect of the drug on learning and memory retention. For the case of higher concentration, embelin at 1.2 mg/kg dose showed a longer escape latency similar to BCCAO group. However, post-training treatment of BCCAO rats with embelin 0.3 and 0.6 mg/kg doses have significantly improved memory consolidation and alleviated the cognitive deficiency caused by chronic cerebral hypoperfusion. Furthermore, embelin has more potential to stabilize memory formed during the consolidation process leading to long-term memory formation thereby overcoming the reference memory deficit induced by BCCAO.

Synaptic transmission and plasticity are the basis of learning and memory; thus, this present study examined whether or not the embelin administration can affect the long-term potentiation (LTP) in hippocampal of BCCAO model. Based on results, synaptic plasticity was found significantly impaired in BCCAO rats, which was in agreement with those of previous studies^12,24,25^. Interestingly, the adverse effect of chronic cerebral hypoperfusion on LTP was significantly attenuated after treatment with 0.3 mg/kg embelin. According to Rong, *et al*.^26^, enhanced LTP in the CA1 and CA3 fields of the dorsal hippocampus were due to a presynaptic mechanism of action. The result of LTP for 0.3 and 0.6 mg/kg was seen parallel with MWM performance supported by the idea that changes in synaptic efficacy underlie learning and memory processes^27^. Meanwhile, in the case of higher concentration, the present results are unusual with embelin 1.2 mg/kg dose displaying lower LTP than that of the BCCAO model. The explanation for the decrease in the LTP for 1.2 mg/kg dose is similar to the previous study using scopolamine-induced memory impairment^17^ in which the compound reached its maximum effect with the decline in cognitive ability at this dose for novel object recognition (NOR) and elevated plus maze (EPM). This experimental result is also supported by the fact that particular drug in which higher doses produce no effect or the opposite effect compared to intermediate doses^28^.

In the case of APP mRNA expression, it was found that chronic cerebral hypoperfused rats displayed significant upregulation due to occlusion of the common carotid arteries. Literature has reported that the chronic cerebral hypoperfusion resulted in an increase in protein level of APP in the hippocampus of rats^29,30^. Meanwhile, the APP mRNA expression was significantly downregulated by the embelin treatment in a dose-dependent manner. Moreover, significant increase in Mapt mRNA level was found in BCCAO rats, which is consistent with the results reported by previous studies^31,32^. Although the Mapt mRNA expression level was significantly decreased by all embelin treatments, the highest dose of embelin at 1.2 mg/kg group showed maximum protection against Mapt in CCH rats. On the other hand, the lowest dose of embelin at 0.3 mg/kg group demonstrated maximum protection against APP. It can be explained from the discrepancy that the downregulation of APP and Mapt by embelin maybe mediated through a different mechanism. This is another interesting outcome as this is the first time that embelin has been reported to downregulate the expression of APP and Mapt, which was directly linked to the AD.

To study the molecular basis of synaptic plasticity impairment, the expression of BDNF and CREB in the hippocampus of BCCAO induced CCH rats was examined. BDNF is a crucial mediator involved in neuronal survival, development and synaptic plasticity^33^. Chronic cerebral hypoperfusion caused by blood flow insufficiency can cause progressive cognitive dysfunction with BDNF and CREB down-regulation. The cAMP‐responsive element binding protein (CREB)-mediated transcription is needed in CNS for neuronal survival^34^. The transcription of the BDNF gene was CREB-regulated in an activity-dependent manner as demonstrated by several studies whereby its expression was involved in neuronal development, synaptic plasticity and neuroprotection^35^. In this present study, BCCAO rats treated with vehicle alone showed a decrease in the BDNF level, which was significantly restored after treated with embelin. Moreover, it was reported that embelin at 0.3 mg/kg was able to enhance BDNF levels of the normal group in CCH rats. Besides, a similar pattern was recorded in CREB expression levels, but was non-significant compared to BCCAO rats treated with vehicle alone. Furthermore, these outcomes were in accordance to MWM and LTP results whereby embelin at 0.3 mg/kg revealed BDNF and CREB1 upregulation as the reason for maximum improvement of spatial memory which is related to hippocampal and enhance the synaptic strength within the CA1 and CA3 neurons. Thus, it can be postulated based upon BDNF and CREB results that embelin is able to activate CREB pathway and improve BDNF mediated synaptic plasticity and neurogenesis.

There are several studies conducted emphasising the pathogenesis of chronic cerebral hypoperfusion and oxidative stress are caused by the implication of oxygen-derived free radicals, which play a significant role in cognitive dysfunction^36^. It has been proven by previous study that embelin has antioxidant properties in the hippocampus of the scopolamine model^17^. Therefore, it can be said that oxidative stress mechanism might have the potential to cause cognitive impairment. SOD in the mitochondrial matrix has been known as the prime line of antioxidant defence system. Hence, the mRNA expression level of SOD1 in the hippocampus was measured to assess whether or not the oxidative stress mechanism is involved in the effect of embelin. Data from this study displayed a good agreement with Zhang, *et al*.^37^ from the observation of a significant decrease in SOD1 mRNA expression level of the BCCAO rats’ hippocampus. Interestingly, administrating embelin has significantly increased the SOD1 mRNA expression level, which indicates the antioxidant action of embelin that might be attributed to a direct receptor-mediated mechanism activating the downstream protein kinase signalling pathways and intracellular antioxidant enzyme systems^25^.

The NFκB was the last gene studied displaying a huge upregulation in the expression level following the CCH, which is in accordance to study reported by Fu, *et al*.^38^. Nuclear factor NFκB is a transcription factor serving a vital role in gene regulation and is applied in inflammation and oxidative stress^39^. In this study, the NFκB expression level in all groups was significantly reduced by embelin treatment.

The glutamatergic, GABAergic, cholinergic and monoaminergic neurotransmitters were observed to highly regulate the hippocampal activity, which play an imperative role in memory acquisition, consolidation and storage in the brain^40,41^. Kaundal, *et al*.^42^ explained that significant alterations in hippocampal neurotransmitters can be observed following BCCAO surgery in rats in addition to cognitive dysfunction. In the present study, BCCAO-induced CCH rats caused a significant elevation in hippocampal glutamate levels, which is in agreement with the results obtained in other AD-like conditions including scopolamine^17,43^ and streptozotocin rat model^20^. On the contrary, the GABA levels in CCH rats were noticed to be slightly decreased but it was statistically non-significant. Moreover, previous studies recorded that imbalance levels in glutamate/GABA have caused excitotoxic neuronal damage and cognitive dysfunction in related neurological disorder^41^, which was similar to the present results. In the current study, embelin treatment significantly reduced hippocampal glutamate level and restored GABA level (non-significant) in CCH rats. It was also discovered that BCCAO rats have caused a reduction in ACh level, thus indicating cognitive impairment due to the metabolism of ACh into acetate and choline by acetylcholinesterase (AChE)^20^. Interestingly, BCCAO rats treated with embelin 0.3 and 0.6 mg/kg significantly attenuated ACh level denoting the acetylcholinesterase inhibitor action of embelin. Moreover, these results are also comparable to probe trial performance in MWM. Furthermore, studies have revealed that a rise in the extracellular level of endogenous ACh by AChE inhibitor treatment can contribute to an increase in cerebral blood flow^12,44,45^. Hence, it can be explained that embelin is neuroprotective in BCCAO-induced CCH rats via the up-regulation of cholinergic function by restoring cerebral blood flow.

**Figure 7:**
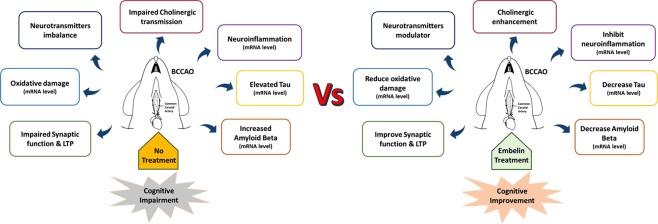
Framework to describe the effectiveness of embelin treatment to improve cognitive impairment compared with no treatment in rat model of Chronic Cerebral Hypoperfusion.

Many studies conducted on knockout animals reported an increase in serotonin level, which improved memory performance, whereas the reduction in this neurotransmitter led to impairment in spatial memory^46^. Therefore, the primary function of serotonin in spatial learning and memory could be due to its involvement in cortical-hippocampal synaptic connections^47^. In this CCH rat model, significant decrement in serotonin level was found in the hippocampal of BCCAO rats compared to sham rats. However, the present results showed that BCCAO rats treated with embelin could not significantly ameliorate the serotonin levels to normal even though there was a slight increment in serotonin level when compared to that of BCCAO rats. Hence, these outcomes suggest that embelin might modulate spatial memory through different mechanisms other than serotonin. Although the exact molecular mechanism of embelin on the expression of neurotransmitters level remained unclear, the results from this study are convincing in postulating that embelin might play a role as a neuromodulator. Thus, the improvement in cognitive functions based on MWM and LTP in this BCCAO-induced CCH rats could be linked to the ability of embelin to modulate hippocampal neurotransmitters to a normal state. In conclusion, this study has provided novel finding where embelin treatment has mitigated the spatial memory and LTP impairment in a BCCAO-induced CCH rat model. Embelin possessed neuroprotective and anti-AD effects that could be mediated by synaptic plasticity, antioxidant, anti-inflammatory, APP and Mapt gene response, cholinergic activity, neurochemical modulation and BDNF-CREB pathway. Thus, this current study suggests that embelin could become a potential therapeutics compound for treating cognitive disorders including VaD and AD. Figure 7 displays the potential mechanism of embelin action in CCH-induced memory impairment in rodents.

## Materials and Methods

### Animals

In-house bred male Sprague Dawley rats weighing 200–300 g and 6–8 week old were obtained from the Animal Research and Service Centre, Universiti Sains Malaysia (USM), Penang, Malaysia. The rodents were kept in cages maintained under standard husbandry conditions (12:12 h light/dark cycle, constant room temperature with no restriction on food and water supply). Prior to the employment of this study, the rats were allowed to acclimatise for one week in the transit house to reduce stress. We confirm that all experiment animal experimentations were performed in accordance with relevant guidelines and regulations that have been approved by USM Animal Ethics Committee with the reference number USM/Animal Ethics Approval/2015/(97) (707).

### Surgery

Permanent bilateral common carotid arteries occlusion (BCCAO) in rats were performed as previously described^6^. Briefly, a mixture of ketamine (80 mg/kg) and xylazine (10 mg/kg) was utilised to intraperitoneally anaesthetise all the rats. For BCCAO surgery, the common carotid arteries were exposed by a surgical cut at the ventral midline. Both common carotid arteries were permanently ligated doubly using a 5/0 silk suture. The skin incision was then closed, while the rats were kept in a well-ventilated room at a temperature of 25 °C. The sham group was subjected to the same method without BCCAO. All the rats were left for two weeks recovery period before being subjected to the Morris water maze and *in vivo* electrophysiology. Rats that subjected to BCCAO surgery are susceptible to seizures, impaired vision and drastic weight loss. Thus, rats that showed any of these characteristics after BCCAO surgery were excluded from this study. The mortality rate for rats that underwent BCCAO surgery in this study was 20%.

### Experimental design and treatment

**Figure 8:**
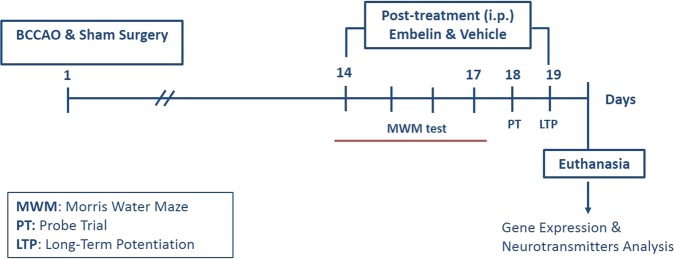
Schematic representation of the experimental procedure and treatment schedule for CCH-induced BCCAO rats.

Embelin preparation and the selection of doses for treatment were determined using the data from our previous study^17^. BCCAO rats were divided into four groups. Group 1: Negative Control (BCCAO + Vehicle) Saline with DMSO (n = 6–7); Group 2: (BCCAO + EMB 0.3 mg/kg) low dose of EMB (n = 6–7); Group 3: (BCCAO + EMB 0.6 mg/kg) medium dose of EMB (n = 6–7); Group 4: (BCCAO + EMB 1.2 mg/kg) high dose of EMB (n = 6–7). The sham rats were labelled as Group 5 (Control) (n = 6–7) and treated with the same vehicle as the negative control. Embelin and vehicle were given intraperitoneally (i.p.) at a volume corresponding to 0.1 ml/100 g of body weight from day 14 onwards. Figure 8 illustrates a schematic representation of the experimental procedure for CCH-induced BCCAO rats.

### Behavioural assessment

#### Morris water maze test

The experiment protocol for the Morris water maze was conducted adopting from Damodaran, *et al*.^12^ with slight modifications. This study selected a black circular pool with 160 cm in diameter and 70 cm in height, which was placed in a test room surrounded by several visual cues. This was then followed by the addition of water into the pool to a depth of 39 cm. Besides, the pool was made opaque by the white paint added to it. The pool was divided into four quadrants, with a platform (10 cm diameter) situated 2 cm below the surface of the water in a fixed position in one quadrant while the opaque water was kept constant temperature at 25 ± 1 °C. Furthermore, the rats were given a pre-training session on the habituation day where they were allowed to swim freely in the pool for 60 s without a platform. All rats were put in the water at four starting points during the training session, respectively, and 60 s was set as the limit for the latency of escaping onto the platform to be recorded as a trial. Four trial were conducted daily within four consecutive days for each rat. Treatments were given intraperitoneally after each training session. It was observed on the fifth day that no platform was present; thus, each rat was subjected to a probe trial. The percentage time spent in the target quadrant was used for obtaining the spatial reference memory for each rat.

#### Long-Term Potentiation (LTP)

The *in vivo* electrophysiological recording was conducted following the protocol proposed by Damodaran, *et al*.^12^. Briefly, urethane was utilised to anaesthetise the rats (2.0 g/kg, i.p., divided into four, 0.5 g/kg doses every 20 min). An incision line was made to expose the skull of the rat on a stereotaxic frame. Standard stereotaxic measurements relative to bregma were utilised as the reference in drilling small holes in their skull to implant the recording electrode in the hippocampus CA1 region (AP: −4.2 mm, mL: −3.0 mm, V: −3.0 mm). The bipolar stimulating electrode was placed into the Schaffer collaterals CA3 region of the hippocampus (AP: −4.2 mm, mL: +3.0 mm, V: −4.0 mm). Meanwhile, to find the stimulating intensity that could evoke 50–60% response of its maximum extracellular field excitatory postsynaptic potentials (fEPSPs) amplitude, stimuli intensities between 0.1 and 1.0 mA with increment of 0.1 mA were delivered to the Schaffer collaterals. The stable baseline was recorded every 30 s for 1 hour. Then, single theta burst stimulation (TBS) comprising of ten bursts (each burst consisting of 5 pulses at 100 Hz) with bursts repeated every 200 ms was delivered to induce LTP. The recording of fEPSPs were recorded every 30 s for 2 hrs.

#### Tissue processing

After the completion of the *in vivo* electrophysiological recording, all the rats were sacrificed under urethane anaesthesia with their brains extracted. Following this, the hippocampi isolation and the samples were stored at −80 °C until further analysis. For gene expression, one part of the hippocampus was transferred into 200μL ice-cold TRIzol®, whereas for neurotransmitter study, the other one was put into 200 μL ice-cold methanol with formic acid.

#### Total RNA extraction and Real-Time PCR

The method of Bhuvanendran, *et al*.^17^ was seen similar to that of this study where the total RNA was extracted with identical Real-time PCR for the CCH- induced rat brain’s hippocampal. The mRNA expression level of genes encoding brain-derived neurotrophic factor (BDNF), superoxide dismutase 1 (SOD1), amyloid precursor protein (APP), microtubule-associated protein tau (Mapt), nuclear factor kappa B (NF-κB) and IMPDH2 (Inosine Monophosphate Dehydrogenase 2) housekeeping gene was measured utilising Applied Biosystem real-time PCR. Threshold cycle (Ct) values of genes of interest was used against the Ct value of housekeeping gene to measure the expression level of five genes of interest using the formula: 2^ (Ct value of housekeeping gene − Ct value of gene of interest).

#### Neurotransmitter analysis using LC-MS/MS

LC-MS/MS was used to estimate the level of neurotransmitters such as glutamate (Glu), γ-aminobutyric acid (GABA), acetylcholine (ACh) and serotonin (5HT) of CCH-induced rats. The protocol for neurotransmitter analysis has been described in details in the previous studies^17^. For validating Glu, GABA, ACh and 5HT, four calibration standards for neurotransmitters in concentration ranges of 250.00–20,000.00, 250.00–20,000.00, 0.25–600.00 ng/mL and 0.05–5.00 ng/mL were used, respectively. The stock for the standards was prepared with methanol (0.1% formic acid) and kept at 4 °C until use. In short, one part of the hippocampus was homogenised in ice-cold methanol containing 1% formic acid followed by vortex-mixed for 60 s. The resulting homogenate was then centrifuged at 14 000 rpm for 10 min at 4 °C. Finally, the resulting supernatant was put into vial inserts for the analysis of LC-MS/MS. ZORBAXE clipse plus C18 RRHD 2.1 × 150.0 mm and 1.8-micron (P/N959759-902) column was utilised to separate the samples, which was then placed on Agilent 6410 Triple Quad (Agilent Technologies, Santa Clara, CA, United States) at 30 °C. The mobile phase comprising 0.1% formic acid in water (Solvent A) and acetonitrile with 0.1% formic acid (Solvent B) was used with a gradient elution of 0–3 min, 50% B; 3–6 min, 95% B; 06–07 min and 95% B at a flow rate of 0.1 ml/min. MS acquisition of Glu, GABA, ACh, and 5HT was done in a positive electrospray ionisation multiple reaction monitoring (MRM) mode.

#### Statistical analysis

Mean ± standard errors of the mean (SEM) were used to express the results obtained. Two-way repeated ANOVA was used to analyse the results of acquisition trial of MWM and differences in fEPSP amplitude after TBS. Statistical analysis for probe trial in MWM,average fEPSP amplitude for 2 h recording, mRNA expression and neurotransmitters levels were analysed using one-way ANOVA. Bonferroni’s post hoc test was used for multiple comparisons. Comparison was made for all the groups with the negative control group (BCCAO) and *p < 0.05, **p < 0.01, ***p < 0.001 and ****p < 0.0001 were set to be statistically significant.The statistical analysis was carried out using GraphPad Prism software (version 7.0).

### Ethics statement

All animal experimentations conducted in this study have been approved by USM Animal Ethics Committee with the reference number USM/Animal Ethics Approval/2015/(97) (707).

## Data Availability

The dataset generated from this study is available from the corresponding authors on reasonable request.
